# Expertise Modulates Neural Stimulus-Tracking

**DOI:** 10.1523/ENEURO.0065-21.2021

**Published:** 2021-08-16

**Authors:** Geoffrey Brookshire, Heather Harden Mangelsdorf, Clara Sava-Segal, Katherine Reis, Howard Nusbaum, Susan Goldin-Meadow, Daniel Casasanto

**Affiliations:** 1 Department of Psychology, University of Chicago, Chicago, 60637, IL; 2 Centre for Human Brain Health, University of Birmingham, Birmingham, B15 2TT, United Kingdom; 3 Department of Psychology, Elmhurst University, Elmhurst, 60126, IL; 4 Department of Psychological and Brain Sciences, Dartmouth College, Hanover, 03755, NH; 5 Center for Cognitive and Social Neuroscience, University of Chicago, Chicago, 60637, IL; 6 Center for Gesture, Sign, and Language, University of Chicago, Chicago, 60636, IL; 7 Department of Human Development, Cornell University, Ithaca, 14850, NY; 8 Department of Psychology, Cornell University, Ithaca, 14850, NY

**Keywords:** electroencephalography, entrainment, sign language, stimulus-tracking

## Abstract

How does the brain anticipate information in language? When people perceive speech, low-frequency (<10 Hz) activity in the brain synchronizes with bursts of sound and visual motion. This phenomenon, called cortical stimulus-tracking, is thought to be one way that the brain predicts the timing of upcoming words, phrases, and syllables. In this study, we test whether stimulus-tracking depends on domain-general expertise or on language-specific prediction mechanisms. We go on to examine how the effects of expertise differ between frontal and sensory cortex. We recorded electroencephalography (EEG) from human participants who were experts in either sign language or ballet, and we compared stimulus-tracking between groups while participants watched videos of sign language or ballet. We measured stimulus-tracking by computing coherence between EEG recordings and visual motion in the videos. Results showed that stimulus-tracking depends on domain-general expertise, and not on language-specific prediction mechanisms. At frontal channels, fluent signers showed stronger coherence to sign language than to dance, whereas expert dancers showed stronger coherence to dance than to sign language. At occipital channels, however, the two groups of participants did not show different patterns of coherence. These results are difficult to explain by entrainment of endogenous oscillations, because neither sign language nor dance show any periodicity at the frequencies of significant expertise-dependent stimulus-tracking. These results suggest that the brain may rely on domain-general predictive mechanisms to optimize perception of temporally-predictable stimuli such as speech, sign language, and dance.

## Significance Statement

Information in speech appears in bursts. To optimize speech perception, the brain tracks these bursts of information with slow rhythms in neural excitability (<10 Hz). Here, we tested whether neural stimulus-tracking depends on participants’ non-linguistic expertise. We recorded electroencephalography (EEG) in participants who were experts in either dance or sign language, while they watched videos of dance or sign language. Our results show that participants’ brain activity more closely tracks the stimulus matching their expertise. These results are difficult to explain by entrainment of endogenous oscillations, because sign and dance are not periodic at the frequencies of expertise-dependent stimulus-tracking. The brain may rely on domain-general predictive mechanisms to optimize perception of temporally-predictable information.

## Introduction

During language comprehension, the brain predicts upcoming phonemes, words, phrases, and semantics (Kuperberg and Jaeger, 2016; Pickering and Gambi, 2018), as well as the timing of upcoming events (Nobre and Van Ede, 2018). How does the brain anticipate when linguistic information will appear? Here we show that domain-general expertise modulates the strength of neural stimulus-tracking in frontal cortex but not in sensory cortex.

When people listen to speech, low-frequency (<10 Hz) neural activity synchronizes to bursts of volume in the sound (Ahissar et al., 2001; Luo and Poeppel, 2007; Peelle and Davis, 2012). This phenomenon, called cortical stimulus-tracking (or entrainment in the broad sense; Obleser and Kayser, 2019), occurs during visual as well as auditory perception. When people watch someone speaking, neural activity in visual cortex synchronizes with motion in the video (Power et al., 2012; Park et al., 2016; Bourguignon et al., 2020). Cortical stimulus-tracking is not limited to speech but also arises when people perceive other structured sequences. For example, brain activity synchronizes with rhythms in music (Doelling and Poeppel, 2015), rhythmically-varying sounds (Henry and Obleser, 2012; Henry et al., 2014), and bursts of visual motion in sign language (Brookshire et al., 2017).

Cortical stimulus-tracking may reflect neural predictions of the timing of upcoming bursts of information. Consistent with this proposal, low-frequency neural activity anticipates events in the stimulus, and is not simply a series of superimposed evoked responses (Park et al., 2018; Doelling et al., 2019; Arabkheradmand et al., 2020). Low-frequency oscillatory phase influences both perceptual sensitivity (Busch et al., 2009; Mathewson et al., 2009, 2012; Henry and Obleser, 2012; Neuling et al., 2012; Ng et al., 2012; Cravo et al., 2013; Spaak et al., 2014; Strauß et al., 2015; Riecke et al., 2018) and neural excitability (Lakatos et al., 2005, 2008; Jacobs et al., 2007; Mathewson et al., 2009; Romei et al., 2012; Zoefel et al., 2018), suggesting that stimulus-tracking may serve to boost perception during informative periods of the stimulus. By synchronizing neural oscillations to a stimulus, the brain may tune attention to relevant moments in time (Schroeder and Lakatos, 2009; Giraud and Poeppel, 2012; Peelle and Davis, 2012; Kayser et al., 2015).

How do different areas of the brain contribute to cortical stimulus-tracking? Tracking in sensory cortex depends largely on low-level characteristics of the stimulus. For example, stimulus-tracking in auditory cortex is driven by acoustic edges (Doelling et al., 2014; compare Zoefel and VanRullen, 2016). Furthermore, tracking of a meaningless visual flicker is strongest over occipital cortex (Mathewson et al., 2009; Spaak et al., 2014; Keitel et al., 2017). By contrast, stimulus tracking in frontal cortex may reflect higher-level processes in addition to low-level stimulus characteristics. Tracking of linguistic chunks like phrases and sentences, for instance, is strongest outside of sensory cortex, with one cluster of activity in the inferior frontal gyrus (Ding et al., 2016). Through these higher-order processes, frontal cortex may provide top-down input to sensory cortex (Park et al., 2015, 2018). Here, we test the hypothesis that frontal involvement in stimulus-tracking depends on expertise with the stimulus being perceived.

In this study, we use electroencephalography (EEG) to contrast the involvement of frontal and occipital regions in cortical stimulus-tracking. Although activity in both regions tracks low-level temporal structure, we hypothesize that stimulus-tracking in frontal cortex depends on expertise with the stimulus, whereas tracking in occipital cortex does not depend on expertise. This hypothesis is motivated by prior findings: at frontal EEG channels, coherence to sign language is stronger in fluent signers than in non-signers; at occipital channels, however, coherence does not depend on whether participants know sign language (Brookshire et al., 2017). Here, we tested this hypothesis by examining the effects of expertise on stimulus-tracking. Frontal cortex could predict the timing of upcoming events using either (1) a language-specific mechanism (Ryskin et al., 2020); or (2) a domain-general mechanism (Pickering and Gambi, 2018). This study distinguishes between these possibilities by comparing participants whose expertise is linguistic (American Sign Language; ASL) with participants whose expertise is non-linguistic (ballet).

## Materials and Methods

### Overview

Participants were experts in either ballet or sign language, and were instructed to remain still and relaxed while they watched videos depicting either ballet or sign language. There was no other task. We recorded EEG and computed coherence between brain activity and the instantaneous visual change (IVC; Brookshire et al., 2017) of the movies. All procedures were approved by the Institutional Review Board of the University of Chicago. In total, the experimental session lasted 60–90 min.

### Experimental design

We designed this study to test for an interaction between participant group (signers, dancers) and stimulus type (videos of sign, videos of dance). Specifically, we predicted that signers would show relatively greater coherence to sign language than to dance, compared with dancers. A difference in the overall levels of expertise between groups (within their chosen domain) could not lead to this interaction. Crucially, none of our hypotheses rely on a main effect of participant group or stimulus type. Differences in experience are therefore a source of Type II error, not a source of Type I error. Any significant interactions that arise would do so despite any differences in experience between the groups. 

**Table 1:** Additional statistical tests of the interactions

Comparison	ANOVA	$\eta_{G}^{2}$	LMER	LMER *β*	BEST
Region by stim. cond. by subj. group	*p* = 0.009	0.04 [0.00, 0.24]	*p* = 0.016	−0.92 [−1.68, −0.15]	−0.58 [−1.0, −0.14]
Frontal: stim. cond. by subj. group	*p* = 0.003	0.13 [0.00, 0.37]	*p* = 0.002	1.02 [0.40, 1.64]	−0.98 [−1.6, −0.37]
Occipital: stim. cond. by subj. group	*p* = 0.7	0.0023 [0.00, 0.13]	*p* = 0.7	0.10 [−0.44, 0.64]	−0.13 [−0.71, 0.41]

Columns show the comparison being tested; *p* value computed in an ANOVA; $\eta_{G}^{2}$ with 95% CI from the ANOVA; *p* value computed from model comparisons in linear mixed-effects models (LMER) after dropping the interaction of interest; median and 95% posterior distribution of the interaction *β* parameter estimate of the LMER model; interaction estimates and 95% CI using Bayesian estimation; stim. cond.: stimulus condition (videos of sign vs dance); subj. group: subject group (dancers vs signers).

This design also protects against order effects in stimulus presentation. Stimulus-order effects would appear as a main effect of stimulus, whereas the predicted two-by-two interaction can only appear if each group shows a specific response to the stimulus within their expertise.

### Participants

We recruited two groups of adult participants: (1) experts in ASL who were not familiar with ballet; and (2) experts in ballet who were not familiar with sign language. Participants were recruited through online postings, fliers at dance studios, and emails to ballet schools and Deaf community mailing lists in the Chicago area. All participants had corrected-to-normal vision and no known history of epilepsy, brain surgery, or traumatic brain injuries. We obtained informed consent before beginning the experiment, and paid participants $20 per hour for their participation.

We recorded data from fluent signers (*N *=* *12) and experts in ballet (*N *=* *19). All fluent signers reported learning ASL before age 5, were either Deaf or hard of hearing, and had no experience practicing ballet. In a prescreening questionnaire, all ballet experts reported having practiced ballet for at least 10 years, and no proficiency with ASL or any other sign language. Demographic data about participants’ age and sex is not available, after the anonymized paperwork containing this information was stolen from a car.

### Stimuli

Participants watched two types of silent videos: (1) storytelling in ASL, and (2) ballet vignettes. All videos had native sampling rates of 29.97 Hz.

ASL videos comprised two stories, 8:41 and 9:48 (min:s) long (total 18.5 min). These videos showed a native speaker of ASL telling a story against a static background (Timm and Timm, 2008).

We recorded videos of short ballet vignettes performed by a classically-trained ballet dancer (12 vignettes; total duration 15.05 min). The camera and background remained still during the videos. These videos were appended and separated by a black screen for 5 s. The ballet vignettes were performed along with music, but sound was removed for stimulus presentation.

To ensure that the timing of the videos was accurately linked to the EEG recordings, a small white square flashed in the corner of the display once every 30 frames of video (out of view of the participant). This flash was registered by a photodiode connected to the EEG amplifier. The area of the flash was covered up by the photodiode, so the flash was not visible to the participants.

All participants watched the videos in the same order: two sign videos followed by the dance videos. After each sign video, there was a brief break during which recording quality was checked and electrodes were re-moistened.

### IVC

To derive a time-series of visual information, we calculated the aggregated visual change between successive video frames. This measure, called the IVC, is computed as the sum of squared differences in each pixel across sequential frames:

$$\text{IVC}(t)=\sum_{i}{\left[x_{i}(t)-x_{i}(t-1)\right]}^{2},$$

where *x* is the gray-scale value of pixel *i* at time *t*. A Python module to compute the IVC is available at https://github.com/gbrookshire/ivc. This measure yields motion peaks that show high agreement with methods using deep neural networks or wired motion tracking (Pouw et al., 2018).

### Analysis of stimulus spectra

Before computing the spectra, the IVC traces for each video were normalized by dividing all values by the standard deviation for that video. Power spectra were computed using Welch’s method. The data were split into overlapping segments (segment length 2.13 s, 2^6^ samples; overlap 1.07 s, 2^5^ samples). A Hanning window was applied to each segment, and the linear trend was removed. Fast Fourier transforms (FFTs) were then computed for each segment. The spectrum for each signal was obtained by averaging across segments within each video.

### Fitting periodic and aperiodic components of stimulus spectra

We tested for oscillatory dynamics in the stimuli using the FOOOF algorithm (Donoghue et al., 2020) in Python (v. 1.0.0; https://fooof-tools.github.io). This analysis was computed for frequencies up to 15 Hz, with a minimum peak height of 2 SDs, peak widths limited to 1–8 Hz, no maximum number of peaks, and a knee term for the aperiodic component. We ran the FOOOF algorithm separately on the average spectra for sign and dance stimuli, with averages weighted by the duration of each stimulus video.

### EEG acquisition and preprocessing

We recorded EEG at 250 Hz using a 128-channel net (Electrical Geodesics). Impedances were reduced to <50 kΩ before participants watched each sign video, and before the dance video. EEG analyses were performed in MATLAB using custom software and the open-source FieldTrip package (Oostenveld et al., 2011). Before any analyses, we excluded electrodes that are likely to be contaminated with strong muscle artifacts (along the face, beneath the ears, and at the base of the neck), leaving 103 channels. Electrode movement artifacts were manually identified and rejected by replacing the tagged region with zeros and applying a 4000-ms half-Hanning taper to each side of the artifact. This procedure was also applied to remove regions of time between dance vignettes. Artifacts from blinks and eye-movements were identified and removed using independent component analysis (ICA). We aligned the IVC to the EEG recordings using the photodiode triggers that appeared once every 30 frames of video. We then used cubic spline interpolation to warp the IVC for each 30 frames of video to the corresponding period of EEG data, simultaneously resampling the IVC from 30 to 250 Hz. EEG signals were re-referenced to the average mastoids before computing coherence.

### Coherence analysis

Brain-stimulus coherence was computed independently for each EEG channel, following our previous work (Brookshire et al., 2017). The IVC and EEG data were filtered into overlapping log-spaced frequency bins using phase-preserving forward-reverse Butterworth bandpass filters. Bins were centered on values from 0.5 to 16 Hz, and included frequencies in the range (0.8 *f*, 1.25 *f*), where *f* is the center frequency *f *=* *2*^n^* for n={−1,−0.5,0,...,4}. The Hilbert transform was used to calculate instantaneous phase and power of each signal. Power was computed as the absolute value of the analytic signal, and phase as the angle of the analytic signal. These instantaneous power and phase estimates were then used to calculate coherence:

$$\text{Coh}=\frac{\left|\sum_{t}\left(e^{i\theta (t)}\sqrt{P_{C}(t)\cdot P_{V}(t)}\right)\right|}{\sqrt{\sum_{t}(P_{C}(t)\cdot P_{V}(t))}},$$

where *t* is the time point, *θ* is the phase difference between the IVC and EEG, *P_V_*is power in the IVC, and *P_C_* is power in the EEG recording (Doelling et al., 2014).

### Statistical analyses

We used a randomization procedure to determine statistical significance of coherence between the IVC and EEG recordings. To obtain a null distribution of coherence, the onset of the IVC was circularly shifted to a randomly selected starting point. This procedure preserves the spectro-temporal characteristics of both signals, but eliminates any relationship between them. For each subject, we computed 100 randomly shifted baselines. Coherence was then computed between the EEG signals and the shifted IVC.

### Cluster-based permutation tests

We used non-parametric cluster-based permutation tests (Maris and Oostenveld, 2007) to control for multiple comparisons while testing statistical significance across all frequencies and EEG channels. These analyses were performed in MATLAB using functions from the open-source FieldTrip package (Oostenveld et al., 2011). Within each combination of stimulus type and subject group, we tested for above-chance coherence by comparing empirical cortico-stimulus coherence to coherence computed after randomly shifting the IVC of the stimulus. Separately in each subject, the true difference between empirical and randomly-shifted coherence was compared with a surrogate distribution (*k *=* *10,000) in which the “empirical” data were randomly selected from the group of empirical and randomly-shifted traces. In each permutation, *t* statistics were computed on the difference between empirical and randomly shifted data using dependent-samples linear regressions. These *t* statistics were computed independently for each frequency and channel. The cluster statistic was computed as the maximum cluster size in each permutation. Samples were identified as a member of a cluster if their individual *t* statistic exceeded the threshold (cluster threshold: α = 0.05, two-tailed; minimum number of channels in a cluster = 2). The *p* value was calculated using the standard FieldTrip functions as the proportion of permuted cluster statistics that were more extreme than the empirical value. To compare across stimulus conditions and subject groups, we computed *z*-scores of empirical coherence against the randomly shifted baseline distribution. This procedure takes into account both the central tendency and the spread of the randomly-shifted data. *Z*-scores were computed separately for each stimulus condition within each subject. To test whether each subject group showed different patterns of coherence to videos of sign versus dance, we performed cluster-based permutation tests on the *z*-scored coherence in each stimulus condition, with cluster membership defined using dependent samples *t* tests. To test whether patterns of coherence to a given stimulus type differed between signers and dancers, we performed the same procedure, but cluster membership was defined using independent samples *t* tests. To test for an effect of expertise (the two-by-two interaction between stimulus type and participant group,) we computed subject-wise differences in *z*-scored coherence to sign versus dance stimuli, and then submitted these differences to a cluster permutation test using independent *t* tests to define cluster membership.

### Regions of interest (ROIs) and frequencies of interest

We examined coherence at two *a priori* ROIs (region of interest) defined in a previous study on cortical coherence to videos of sign language (Brookshire et al., 2017): one frontal ROI and one occipital ROI. We defined frequencies of interest based on the peak of coherence in the same previous study, and averaged coherence from frequency bins centered on 0.5–2 Hz. These regions and frequencies of interest were defined before any data analysis or visualization.

Inferential statistics were computed on *z*-scored coherence (against the randomly-shifted null distribution) using R (R Core Team, 2018).

To test whether the two subject groups showed different patterns to sign and dance, and to test for an interaction of expertise by region (frontal/occipital channels), we used within-subjects ANOVAs with type 2 sum of squares, with region and stimulus condition as within-subjects factors, and subject group as a between-subjects factor. Effect sizes for these interactions were computed as $\eta_{G}^{2}$ (Bakeman, 2005) using the ezANOVA function in the *ez* package, and 95% confidence intervals (CIs) of $\eta_{G}^{2}$ were computed using the η_squared function from the *effectsize* package.

We repeated these analyses using linear mixed-effects regressions with maximal random-effects structure, using the lmer function in the *lme4* package; *p* values were computed by dropping the interaction from the model, and performing a likelihood ratio test on the two models. We computed the 95% posterior distribution (10,000 simulations) of the parameter estimates (a second measure of effect size) using the sim function from the *arm* package.

Finally, for a third measure of effect size and variability, we tested these interactions with Bayesian estimation using the BEST procedure implemented in the bayes.t.test function from the *BayesianFirstAid* package. The output of these tests provides a natural effect size: the strength of the interaction in standardized units. For the two-by-two interactions of stimulus condition by subject group (run separately in each ROI), we computed the difference in coherence to videos of sign minus videos of dance (separately for each subject), and then performed Bayesian estimation on these difference scores between signers and dancers. This differencing procedure accounts for the within-subjects variance in our design. For the two-by-two-by-two interaction of region, stimulus condition, and subject group, we performed a similar procedure. Within each region, we obtained the difference between coherence to videos of sign minus videos of dance (separately for each subject). We then computed the difference of those differences across regions (frontal minus occipital), and used bayes.t.test to test for an effect of subject group on this difference of differences.

For pairwise comparisons between coherence to dance and sign stimuli, we used within-subjects Welch’s *t* tests. For pairwise comparisons between signers and dancers, we used two-sample Welch’s *t* tests. We tested whether individual conditions (e.g., signers watching videos of sign) showed above-chance coherence using one-sample Welch’s *t* tests. We supplemented these *t* tests with non-parametric one-sample and two-sample Wilcoxon tests using the wilcox.test function. For *t* tests, effect sizes and 95% CIs were computed as Cohen’s *d* using the cohens_d function in the *rstatix* package. We also computed effect sizes and 95% CIs as Hedges’s *g*, using the cohen.d function from the *effsize* package. Finally, we computed Bayesian estimates and credible intervals using the BEST procedure implemented in the bayes.t.test function from the *BayesianFirstAid* package.

### Code accessibility

All data and code used in the experiment will be made available on request. Analyses were run using MATLAB, Python, and R (specific analyses detailed above). The main EEG analyses were run on a Linux computing cluster (“Acropolis” at the University of Chicago), and artifact rejection and the region-specific and frequency-specific statistics were run on a desktop running Ubuntu 18.04.5 LTS.

## Results

We used EEG to measure stimulus-tracking in human participants who were experts in either ballet dancing or sign language. Participants watched silent videos of ballet and sign language, and we quantified stimulus-tracking using cortico-stimulus coherence. If stimulus-tracking depends on domain-general predictive processes in frontal cortex, then coherence at frontal channels (but not occipital channels) should depend on expertise, with signers showing stronger coherence to sign language and dancers showing stronger coherence to dance.

### Temporal structure in sign and dance

**Figure 1.**
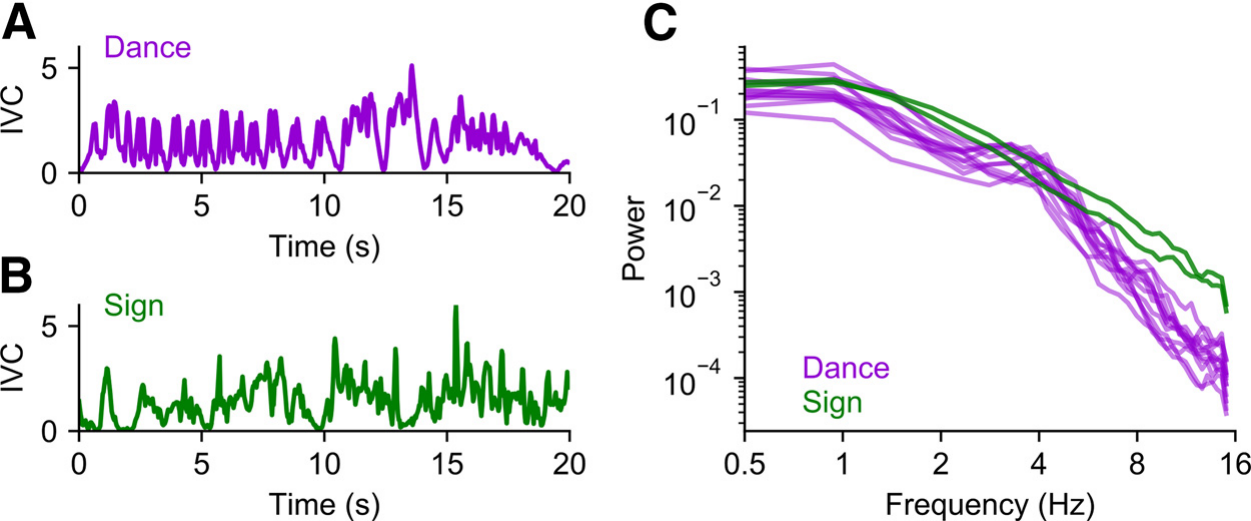
Temporal structure in dance and sign language. ***A***, Example time course of 20 s of the IVC (instantaneous visual change) of a dance video. IVC has been standardized by dividing out its standard deviation. ***B***, Example time course of 20 s of the IVC of a sign language video. ***C***, Spectra of dance and sign stimuli. Each trace shows a separate stimulus video. Dance videos: 12 segments, 15.05 min in total. Sign videos: 2 segments, 18.50 min in total.

We quantified visual information in the sign and dance stimuli using the IVC, a measure of aggregated pixel-wise change (Brookshire et al., 2017; Pouw et al., 2018). Although neither dance nor sign language was strongly rhythmic, the IVC of dance displayed brief periods of repeated segments (Fig. 1*A*,*C*). The IVC of sign language, in contrast, did not show clear oscillatory activity at any timescale (Fig. 1*B*,*C*). We examined periodic and aperiodic structure in these stimuli using the FOOOF algorithm (Donoghue et al., 2020). This analysis confirmed that the dance stimuli show weak periodicity in the θ-band (peak 4.4 Hz; peak height 0.29; bandwidth 1.6 Hz; *R*^2^ = 0.992). Sign language, by contrast, did not display any periodic components (*R*^2^ = 0.996).

### Coherence at frontal but not occipital channels depends on expertise

To test whether neural stimulus-tracking depends on expertise, we computed cortico-stimulus coherence to these videos of ballet and sign language. We examined coherence in two groups of participants: (1) fluent signers of ASL who had no experience with dance (signers), and (2) expert ballet dancers who had no experience with any sign language (dancers).

As an initial test for effects of expertise, we used cluster-based permutation tests to determine whether coherence varies between subject groups and stimulus conditions. These analyses considered activity at all frequencies and across all EEG channels.

**Figure 2.**
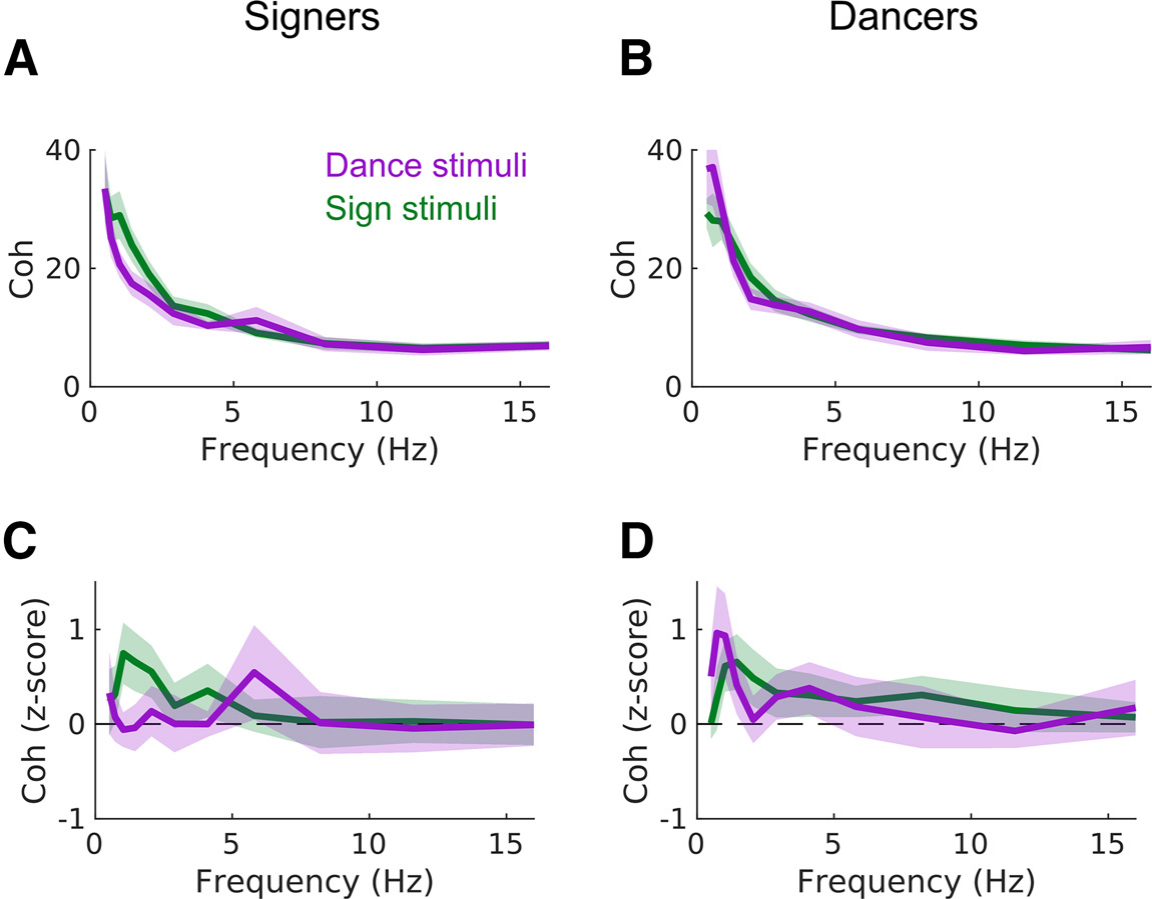
Expertise modulates cortico-stimulus coherence. Coherence spectra averaged over all EEG channels. ***A***, Raw coherence in fluent signers, shown separately for videos of dance and sign. Because cortico-stimulus coherence values depend on the number of pixels in the video, coherence is displayed in arbitrary units (a.u.). ***B***, Raw coherence spectra in expert dancers. Details as in ***A***. ***C***, *Z*-scored coherence (empirical vs randomly shifted) in fluent signers. ***D***, *Z*-scored coherence in expert dancers. In all panels, the line shows the average coherence spectrum across subjects and EEG channels, and the shaded area shows the 95% CI across each subject’s average.

We directly tested for effects of expertise by computing the difference in coherence to videos of sign versus dance, separately within each participant, and then comparing these differences between signers and dancers. This analysis revealed a significant effect of expertise on coherence (*p *= 0.001). Signers showed stronger coherence to sign than to dance (*p *= 0.0004), with above-chance coherence to sign (*p *= 0.0001) but not to dance (no clusters found; Fig. 2*A*,*C*). In contrast, dancers showed stronger coherence to dance than to sign (*p *= 0.03), with above-chance coherence both to sign (*p *= 0.0001) and to dance (*p *= 0.0001; Fig. 2*B*,*D*). These tests reveal that cortico-stimulus coherence depends on expertise, with each group showing stronger stimulus-tracking to the stimulus matching their expertise. These results cannot be accounted for by other known differences between signers and dancers (such as cortical reorganization in deaf participants; Finney et al., 2001); other differences between groups would predict a main effect of subject group, but not an interaction between subject group and stimulus type.

**Figure 3.**
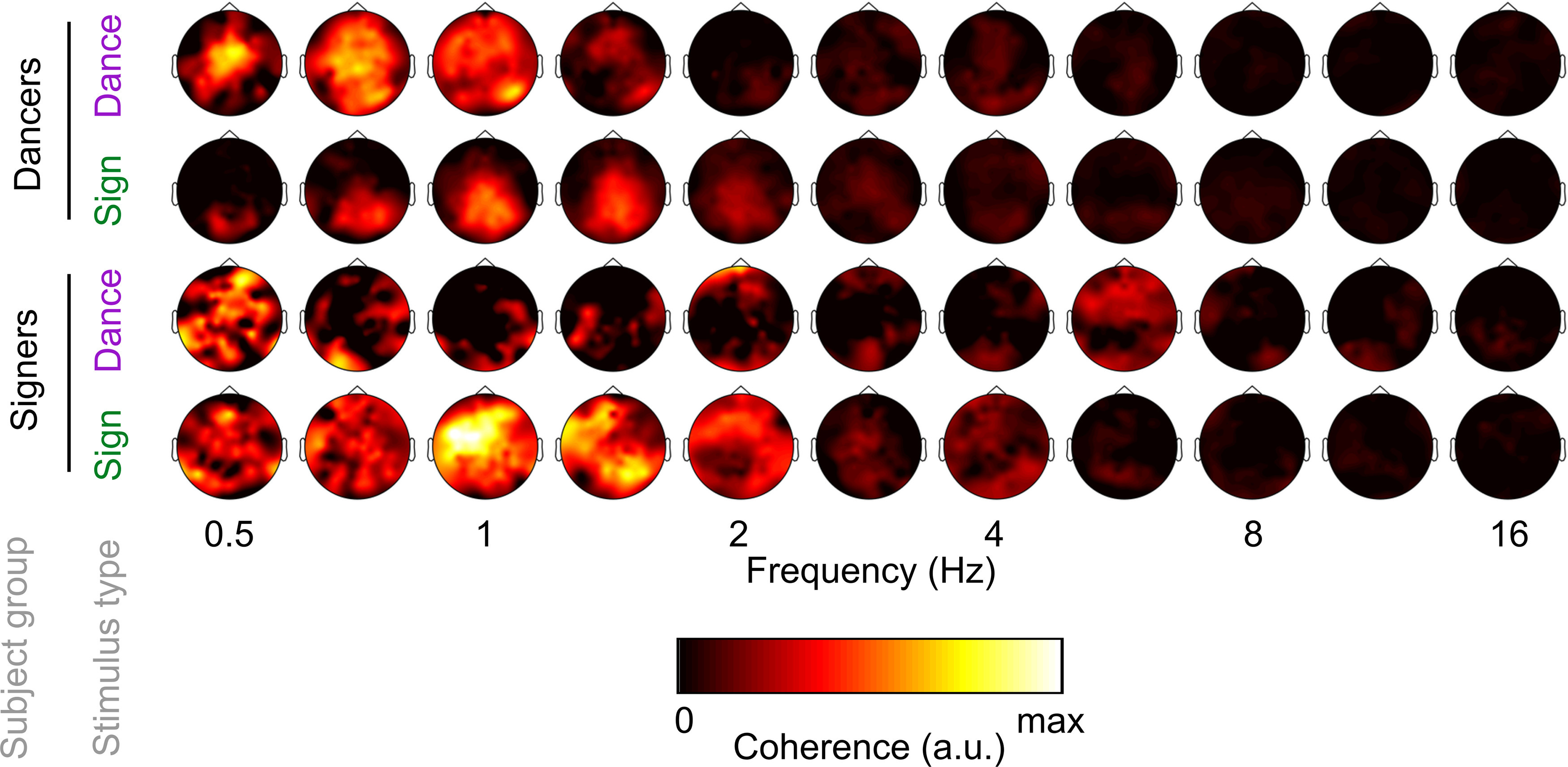
Scalp topographies of cortico-stimulus coherence by subject group, stimulus type, and frequency. All topographies are plotted on the same color scale, and depict raw coherence. Because cortico-stimulus coherence values depend on the number of pixels in the video, coherence is displayed in arbitrary units (a.u.).

Examining the scalp topography of coherence across conditions, fluent signers showed robust coherence to videos of sign language, peaking around 1 Hz over a broad area of central, frontal, and occipital channels (Fig. 3, bottom row). Signers did not show clear topographies of coherence to dance at any frequencies. In contrast, dancers showed strong coherence to videos of ballet, peaking between 0.5 and 1 Hz at central channels (Fig. 3, top row). Dancers showed only moderate coherence to videos of sign language, centered over occipital channels around 1 Hz.

To further investigate how expertise influences cortico-stimulus coherence, we explicitly contrasted coherence from 0.5 to 2 Hz at frontal and occipital ROIs. These frequencies and regions were selected a priori (Materials and Methods). This frequency band captures modulations at the rate of short phrases and slow signs in ASL (Bellugi and Fischer, 1972; Hwang, 2011). We performed these analyses on *z*-scores of empirical coherence against the randomly shifted baseline.

**Figure 4.**
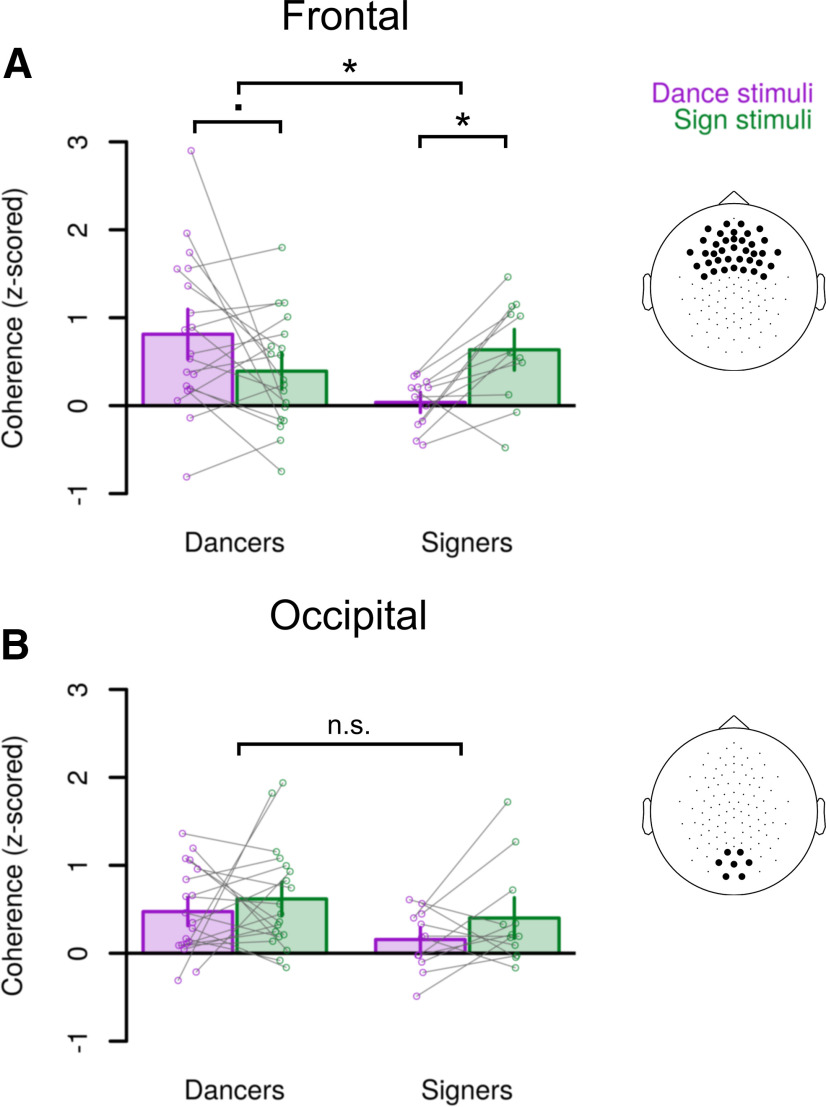
Coherence at 0.5–2 Hz depends on expertise. ***A***, *Z*-scored coherence (empirical vs randomly shifted) at the frontal ROI (region of interest). Channels included in the *a priori* ROI are highlighted in the layout on the right. ***B***, As in ***A*** but averaged over the occipital ROI. Bar heights show the average coherence, and error bars show within-subjects standard errors (Morey, 2008). Points show coherence in individual participants, and lines connect observations from the same participant; *p < 0.005, .*p *<* *0.1; n.s. (not significant) *p *>* *0.1.

At frontal channels, signers and dancers showed different patterns of coherence to sign and dance (*F*_(1,29)_ = 10.9; *p *= 0.003; Fig. 4*A*; Table 1). Signers showed stronger coherence to sign than to dance (*t*_(11)_ = – 3.6; *p *= 0.004; Table 2), with above-chance coherence to sign (*t*_(11)_ = 3.9; *p *= 0.003) but not to dance (*t*_(11)_ = 0.45; *p *=* *0.66). Dancers, in contrast, did not show a significant difference in coherence to sign and dance (*t*_(18)_ = 1.9; *p *= 0.07), although the numerical difference trended in the predicted direction of stronger coherence to dance than to sign. Dancers showed above-chance coherence to both dance (*t*_(18)_ = 4.0; *p *= 0.0008) and to sign (*t*_(18)_ = 2.7; *p *= 0.01). Coherence to dance was stronger in dancers than in signers (*t*_(23.4)_ = 3.6; *p *= 0.002). This analysis did not reveal a statistically significant difference in coherence to sign between signers and dancers (*t*_(25.5)_ = – 1.1; *p *= 0.28), although the difference trended in the predicted direction, with signers showing numerically stronger coherence to sign than dancers.

**Table 2:** Additional statistical tests of the pairwise and within-sample effects

ROI	Comparison	Wilcoxon	BEST 95% CI	Hedges’s *g*	Cohen’s *d*
Frontal	Dancers: stim. cond.	V = 135, *p* = 0.11	0.36 [−0.12, 0.83]	0.52 [−0.07, 1.11]	0.473 [0.005, 0.83]
	Signers: stim. cond.	V = 7, *p* = 0.009	−0.61 [−0.99, −0.23]	−1.21 [−2.14, −0.28]	−1.03 [−2.35, −0.39]
	Dance: subj. group	W = 185, *p* = 0.003	0.75 [0.29, 1.2]	1.06 [0.28, 1.84]	1.19 [0.65, 1.99]
	Sign: subj. group	W = 89, *p* = 0.3	−0.26 [−0.75, 0.24]	−0.39 [−1.13 0.35]	−0.40 [−1.39, 0.31]
	Dancers watching dance	V = 178, *p* = 0.0003	0.79 [0.37, 1.2]	0.89 [−0.08, 1.85]	0.93 [0.56, 1.45]
	Dancers watching sign	V = 152, *p* = 0.02	0.38 [0.074, 0.70]	0.59 [−0.35, 1.53]	0.62 [0.21, 1.14]
	Signers watching dance	V = 44, *p* = 0.7	0.046 [−0.15, 0.24]	0.12 [−1.05, 1.29]	0.13 [−0.48, 0.94]
	Signers watching sign	V = 74, *p* = 0.003	0.65 [0.24, 1.0]	1.03 [−0.22, 2.28]	1.12 [0.54, 2.64]
Occipital	Dancers: stim. cond.	V = 84, *p* = 0.7	−0.094 [−0.49, 0.29]	−0.25 [−0.94, 0.43]	−0.17 [−0.59, 0.4]
	Signers: stim. cond.	V = 24, *p* = 0.3	−0.24 [−0.63, 0.15]	−0.48 [−1.18, 0.23]	−0.43 [−1.1, 0.15]
	Dance: subj. group	W = 154, *p* = 0.1	0.31 [−0.03, 0.64]	0.70 [−0.05, 1.46]	0.75 [0.14, 1.47]
	Sign: subj. group	W = 145, *p* = 0.2	0.23 [−0.22, 0.69]	0.36 [−0.38, 1.10]	0.37 [−0.4, 1.3]
	Dancers watching dance	V = 174, *p* = 0.0006	0.47 [0.23, 0.73]	0.91 [−0.05, 1.88]	0.95 [0.58, 1.49]
	Dancers watching sign	V = 184, *p* = 0.00005	0.59 [0.30, 0.89]	0.99 [0.02, 1.97]	1.04 [0.75, 1.53]
	Signers watching dance	V = 56, *p* = 0.2	0.16 [−0.066, 0.39]	0.43 [−0.76, 1.62]	0.47 [−0.1, 1.39]
	Signers watching sign	V = 71, *p* = 0.009	0.34 [−0.001, 0.72]	0.65 [−0.55, 1.86]	0.71 [0.44, 1.22]

Columns show the ROI; the comparison being tested (e.g., dancers: stim. cond. is the effect of stimulus condition within dancers); non-parametric Wilcoxon tests; Bayesian estimates with 95% credible interval; Hedges’s *g* with 95% CI; Cohen’s *d* with 95% CI.

The effect of expertise on coherence differed between frontal and occipital channels (*F*_(1,29)_ = 7.8; *p *= 0.009; Table 1). At occipital channels, signers and dancers did not show different patterns of coherence to sign and dance (*F*_(1,29)_ = 0.13; *p *=* *0.72; Fig. 4*B*; Table 2). Dancers showed above-chance coherence to both dance (*t*_(18)_ = 4.2; *p *= 0.0006) and sign (*t*_(18)_ = 4.5; *p *= 0.0003), and signers showed above-chance coherence to sign (*t*_(11)_ = 2.5; *p *= 0.03) but not to dance (*t*_(11)_ = 1.6; *p *= 0.13). There was no statistically significant difference in occipital coherence between videos of sign and dance (*F*_(1,29)_ = 1.9; *p *=* *0.18; $\eta_{G}^{2}=0.032$), or between signers and dancers (*F*_(1,29)_ = 4.0; *p *= 0.054; $\eta_{G}^{2}=0.063$).

These findings suggest that temporal predictions are generated in frontal cortex based on expertise with the stimulus. Because of the poor spatial resolution of EEG, these data do not allow for precise localization of these different patterns of activity. However, these results are consistent with prior studies in demonstrating that frontal cortex generates predictions of upcoming events (Dürschmid et al., 2019).

We find that the critical interactions are significant (or the confidence intervals on the parameter estimate do not include zero) when measured using ANOVAs, mixed-effects regressions, and Bayesian estimation (Table 1). However, the confidence intervals on $\eta_{G}^{2}$ for these interactions do include zero. We suggest that this may occur because the $\eta_{G}^{2}$ confidence intervals do not fully account for the within-subjects structure of the data.

## Discussion

In this study, we found that stimulus-tracking at frontal channels depends on expertise, whereas stimulus-tracking at occipital channels does not. Frontal activity from 0.5 to 2 Hz more closely synchronizes with the stimulus when people are experts in what they are perceiving. Fluent signers showed stronger frontal coherence to videos of sign than to videos of ballet, whereas expert ballet dancers showed stronger coherence to videos of ballet than to videos of sign language. Occipital activity, however, robustly tracked the videos regardless of whether they matched participants’ expertise. These results suggest that frontal cortex is preferentially involved in generating sensory predictions during stimulus-tracking.

### Entrainment versus flexible stimulus-tracking

Our results are unlikely to be driven by entrainment in the narrow sense (Lakatos et al., 2019; Obleser and Kayser, 2019), in which ongoing, endogenous cortical oscillations align with oscillations in the stimulus. We show that dance is only weakly periodic, and sign language may not be periodic at all. Instead, both sign and dance are quasi-periodic, similar to speech (Rimmele et al., 2018). This lack of strong periodicity makes it unlikely that the brain tracks motion in sign and dance by entraining endogenous neural oscillations. Furthermore, if stimulus-tracking is driven by neural entrainment to external rhythms, then we would expect to see the strongest entrainment in sensory areas, in which the stimulus is most faithfully represented. Instead, we find that expertise only modulates stimulus tracking outside of sensory cortex. Together, these considerations suggest that temporal predictions in frontal cortex may flexibly adjust to temporal structure in the stimuli.

### Expertise and attention

We suggest that expertise boosts stimulus-tracking by enabling participants to more accurately predict upcoming changes in the stimulus. Could these results instead derive from differences in how participants attend to stimuli they are familiar with? When people attend to a stimulus, it elicits stronger responses in the brain (Treue, 2001). In fact, attending to a stimulus also boosts cortical stimulus-tracking (Kerlin et al., 2010; Zion Golumbic et al., 2013; O’Sullivan et al., 2015). If participants preferentially attended to the stimulus they were more familiar with, we would expect stronger coherence for the stimuli matching their expertise. Could differences in attention explain our results? In prior studies, the effects of attention on stimulus-tracking are strongest in sensory cortex (Kerlin et al., 2010; Zion Golumbic et al., 2013; O’Sullivan et al., 2015). In contrast, we find an effect of expertise *only* at frontal channels, with no difference at occipital channels. Although we cannot definitively rule out expertise-related differences in attention, it is not clear why an attention-based effect would arise in frontal but not sensory cortex.

### The role of frontal cortex in stimulus-tracking

Our results build on the conclusions of prior studies, which suggest that frontal and motor cortex may coordinate temporal predictions by providing top-down modulatory input to sensory cortex. Although cortical stimulus-tracking is often strongest over sensory cortex, it also occurs in frontal cortex (Molinaro et al., 2016; Park et al., 2016; Brookshire et al., 2017). During stimulus-tracking, frontal areas modulate phase in auditory cortex in both the δ (1–3 Hz) and θ (4–7 Hz) bands (Park et al., 2015, 2018). When people perceive someone speaking, θ-band activity in motor cortex synchronizes to auditory cortex (Assaneo and Poeppel, 2018), and drives activity in visual cortex (Hauswald et al., 2018). Stimulus-tracking in sensory cortex also depends on the power of α and β oscillations in frontal cortex (Kayser et al., 2015; Keitel et al., 2017; Morillon and Baillet, 2017), and frontal neurodegeneration disrupts prediction-related beta activity during speech perception (Cope et al., 2017). Our findings extend this literature by showing that stimulus-tracking in frontal cortex depends on expertise with the stimuli at hand.

Some researchers posit that frontal stimulus-tracking reflects top-down influence on visual and auditory cortex (Park et al., 2015; Brookshire et al., 2017; Hauswald et al., 2018). However, we do not find any expertise-linked modulation of visual cortex, despite the observed differences in frontal cortex (see also Brookshire et al., 2017). Perhaps low-frequency frontal activity modulates higher-frequency visual activity; future studies using methods with improved spatial resolution could test how stimulus-specific temporal predictions in frontal cortex modulate activity in sensory cortex.

### Language-specific or domain-general mechanisms?

What information does frontal cortex use to guide temporal predictions during stimulus-tracking? Some researchers hypothesize that neural synchronization involves processes that are specific to oral speech (Molinaro and Lizarazu, 2018), and that linguistic predictions may rely on language-specific predictive mechanisms (Ryskin et al., 2020; Shain et al., 2020). In contrast, we show here that stimulus-tracking depends on domain-general mechanisms: whatever mechanism supports expertise-dependent synchronization, it operates over both sign language and dance. This conclusion is consistent with prior studies on cortical stimulus-tracking. Cortical activity synchronizes with rhythms in music, and this synchronization is stronger in experts with more musical training (Doelling and Poeppel, 2015; Harding et al., 2019). Furthermore, when people listen to complex syncopated rhythms, neural activity synchronizes with the imagined pulse underlying the rhythm; this synchronized activity is stronger in expert participants who can more accurately tap along to a beat (Tal et al., 2017). Although results such as these are often assumed to reflect entrainment with endogenous oscillations, tracking of rhythmic stimuli may partly rely on a non-oscillatory mechanism; stimulus-tracking also occurs when there are no consistent oscillations in either the stimulus (Daume et al., 2021) or in brain activity (Breska and Deouell, 2017).

Similar results appear in studies using fMRI; in regions associated with speech perception, listening to music evokes stronger BOLD responses in expert violinists than in non-musicians (Dick et al., 2011). These convergent findings indicate that cortical stimulus-tracking depends at least in part on domain-general expertise.

### Coherence to subvocalized descriptions of dance?

Could our results be accounted for by a language-specific mechanism coupled with subvocalized narration of the dance videos? Individual dance movements often have conventionalized names. In theory, dancers could show greater coherence to dance because they subvocally rehearse the names of each movement. However, this account is not consistent with prior findings about the cognitive and neural basis of dance perception. Movements in dance are complex, and can differ in their movement quality (floating, slashing, etc.), weight (light, strong), time (sustained, sudden), and degree of spatial focus (direct, indirect; Groff, 1995; Warburton et al., 2013). To result in significant cortico-stimulus coherence, subvocalized speech would need to precisely align with the time course of movement in the dance videos. Furthermore, neuroimaging experiments suggest that instead of subvocalizing the name of each movement, dancers covertly perform motor imagery when they watch dance, leading to activation in motor networks that depends on dancers’ experience performing the specific movements being perceived (Calvo-Merino et al., 2005, 2006; Cross et al., 2006, 2009; Orgs et al., 2008; Bläsing et al., 2012). In summary, our findings and prior results are not consistent with a language-specific mechanism operating over verbal labels of the dance movements. Instead, our results are consistent with the proposal that perception of dance movements involves covert motor simulations.

### What aspects of the stimuli drive synchronization?

What features of the stimuli does brain activity lock onto? We find that cortical coherence to sign and dance is strongest around 1 Hz, despite the fact that the IVC of dance is periodic around 4.4 Hz, and individual signs appear at ∼2–2.5 Hz (Bellugi and Fischer, 1972; Hwang, 2011). We propose that brain activity synchronizes to higher-level chunks of movement in sign and dance, because of the higher temporal predictability of these larger chunks. This proposal is consistent with findings in neural tracking of auditory speech. Although syllables and fluctuations in the volume of speech occur at ∼2–10 Hz (Greenberg et al., 2003; Chandrasekaran et al., 2009; Ding et al., 2017), cortex often synchronizes to speech most strongly at lower frequencies (0.5–4 Hz; Luo et al., 2010; Bourguignon et al., 2013; Gross et al., 2013; Park et al., 2015; Mai et al., 2016; Molinaro et al., 2016; Keitel et al., 2017; Molinaro and Lizarazu, 2018). Instead of synchronizing to individual syllables, cortical activity may synchronize to prosodic fluctuations in speech (Bourguignon et al., 2013; Keitel et al., 2017) or to short units such as phrases. The brain may lock onto predictable chunks in dance and sign language that are analogous to short phrases in speech.

### Why don’t signers show above-chance coherence to dance?

At frontal channels, we found that dancers show above-chance coherence to both sign and dance, whereas signers show above-chance coherence only to sign (but not to dance). What causes the lack of significant coherence to dance in signers?

First, this could reflect a difference between the participant groups. Perhaps dancers have learned to treat all sorts of body movements as potential dances, allowing their brains to track the unfamiliar movements of sign language. Alternatively, signers may have learned to specifically process movements with linguistic content, causing their brains to be more “selective” about the movements they follow.

Second, signers’ lack of coherence to dance may reflect a difference between the sign and dance stimuli. Perhaps sign language has some characteristics that facilitate stimulus-tracking even in non-signers (e.g., more predictable kinematics), whereas experience is required to enable stimulus-tracking of dance. Further research is necessary to determine the factors that give rise to this pattern of results.

### Conclusion

In conclusion, we find that cortical stimulus-tracking at frontal channels is modulated by expertise, whereas stimulus-tracking at occipital channels is not. By flexibly adjusting low-frequency neural activity, networks in frontal cortex may align periods of increased excitability with bursts of information in the stimulus.

## Synthesis

Reviewing Editor: Christina Zelano, Northwestern University Decisions are customarily a result of the Reviewing Editor and the peer reviewers coming together and discussing their recommendations until a consensus is reached. When revisions are invited, a fact-based synthesis statement explaining their decision and outlining what is needed to prepare a revision will be listed below. The following reviewer(s) agreed to reveal their identity: Leon Franzen.

Both reviewers agreed that this study is interesting and novel and has the potential to contribute valuable information to the field. However, both reviewers raised concerns about the statistical methods which will need to be addressed before the manuscript can be considered further. See detailed comments and suggestions about the statistics in the original reviewers comments below. Pay close attention to Reviewer 1’s comments and suggestions about statistics. During discussions, both Reviewers agreed on the statistical concerns raised by Reviewer 1. Regarding the comment about source reconstruction, if the authors do not have individual structural MRIs from the participants, this may not be possible to address, and is not completely necessary. However all other statistical concerns should be addressed.

Reviewers original comments:

Reviewer 1:

Thank you for the opportunity to review your manuscript “Expertise modulates neural stimulus tracking”. In this concisely written manuscript the authors report an investigation of cortical tracking in response to videos of sign language and ballet dance by experts in either skill using coherence between the visually presented content and electrophysiological activity in two hypothesis-driven clusters over frontal and occipital cortex. While I particularly like the originality of the idea of this study, I do have concerns with respect to the statistical methodology and interpretation of the results, which I’m outlining below. In my assessment the performed analyses are in part unclear and fall short of corroborating the, in part, strong claims.

Major points

Introduction

1. Given the specific subject groups/stimuli of the manuscript and the role of bottom-up and top-down processing in endogenous oscillations mentioned in the introduction, I’d have expected more background on specific differences that are intrinsic to the processing of the two used types of stimuli, such as sign language in deaf and hard of hearing individuals potentially leading to the coherent activation of language networks (including frontal areas) while a motor/athletic skill such as ballet dance may lead to visualizing or mentally performing movements during the viewing of dance videos. It may also be conceivable that ballet dancers actually infer the rhythm of the music that underlies the dance performance they are watching. However, information on such background or links to it are currently absent from the introduction. I suggest that the authors reflect on how to incorporate these aspects in their theoretical motivation of the study, particularly as these are discussed in later on in the discussion.

2. Lines 65-75: I was uncertain about being able to follow the argument that despite reports showing that frontal cortex drives stimulus alignment in sensory areas the authors expect opposite effects of coherence between occipital and frontal clusters of electrodes given a viewer’s expertise. It would help if the authors could clarify their argument that stimulus tracking in frontal cortex reflects higher-level processes and how this is linked to findings regarding expertise. This is currently not mentioned in the introduction. Elaborating on the background, particularly when bridging different topics and fields, would help the reader being able to follow the argument and the hypotheses that are presented as a consequence of it.

Methods

3. While it can be assumed that individuals who are deaf or hard of hearing engage and practice sign language almost on a daily basis, it remains unclear how often the recruited ballet dancers have practiced ballet. Did the authors collect more information on their frequency of practicing ballet or other indicators that are suggestive of individual level expertise? This could lead to substantial differences in expertise between the two groups.

4. Line 119: I presume there may be order effects due to the presentation of the videos in the same order without counterbalancing across participants in a rather long experiment. Given that the experiment lasted for at least 60 minutes, I’d like the authors to reflect on potential order and fatigue effects that have been introduced by their design. It could be that dancers were more fatigued when watching the ballet videos and signers less attentive during this second/later part of the experiment. While nothing can be done about this in the presented study, this should be acknowledged in the limitations.

5. Line 177 & 194: Was the mean, median, or 95% confidence interval of these 100 shifted baselines used to establish the one baseline of coherence that was used for comparisons in subsequent parts of the analysis? Further, I suggest to use the median of a higher number of random shifts/iterations for example 500 or 1000 to obtain more robust estimates of the null distribution.

6. Line 187: computing many t-statistics independently for each channel and frequency inflates the risk of type I errors as a consequence of the multiple comparisons problem and the redundancy in the signal of neighbouring EEG channels. You do mention the low spatial resolution of EEG later on in the manuscript.

7. Lines 189-191: I’m uncertain about the exact nature of the cluster statistic mentioned in. The specifics of this method need to be described more clearly as they determine the empirically determined minimum number of channels required to show significant differences to be considered significant (line 191). Given the authors’ approach of clustering channels into two ROIs later on, I’m confused as to why this analysis was performed for each channel separately without taking spatial adjacency into account. I suggest that the authors clarify the description of this part of their statistical methodology and consider an approach that uses information from adjacent electrodes/channels (and temporal samples) simultaneously in the computation of a maximum cluster size using t-statistics to correct for multiple comparisons, as implemented in the LIMO (Pernet et al., 2011) and other openly accessible toolboxes.

8. Line 191: How was the p-value calculated exactly? What exactly constitutes the empirical value here? A t-statistic, the 95% confidence interval? This remains unclear.

9. Line 201: In this section, it remains unclear what cluster membership was determined exactly. Generally, the approach of testing a 2x2 interaction by comparing the within-subjects difference scores directly should be improved. Given the rather small sample size and likely individual differences in coherence, I’d suggest to employ linear mixed-effects models, as implemented in the R package lme4 (Bates et al., 2015) among others, which consider all relevant main effects and interactions simultaneously while also accounting for variation introduced by the specific sample and participants. For example, these models do not assume that all subjects in an experimental group showed closely resembling coherence.

10. Line 214-223: Due to the nature of t-tests comparing point estimates of central tendency (means) that are easily affected by outliers, this approach cannot be considered robust. I’d suggest to make use of the full 95% confidence interval by comparing whether this includes 0 or not. As well, since Cohen’s d can be liberal and biased, these t-tests should be complemented with robust effect sizes and their 95% confidence interval, such as (a bootstrapped) Hedges’ g as implemented in Matlab (Hentschke & Stüttgen, 2011) or other software.

11. Lastly, please see my earlier comment on the advantages of mixed-models in the context of the present design, which I would invite the authors to use instead of ANOVAs. The application of the outlined methodological improvements would give the reader more trust in the results and a better ability to judge the magnitude of the effects.

Results

12. Lines 227, 258 and 293: The claim of the paper is that the mechanism found here relies on domain-general predictive processes. For being able to draw this conclusion, authors outline here that signers are supposed to show stronger coherence to sign language and dancers to dance at frontal channels but not occipital channels. My main concern is that the results presented in line 258 do not warrant the univocal conclusion the authors draw in the remainder of the manuscript because this pattern of results holds only true for signers but not for dancers. The latter group showed above chance coherence to both types of videos (p = .0001). I don’t think that this result supports the main claim that “These tests reveal that cortico-stimulus coherence depends on expertise, with each group showing stronger stimulus-tracking to the stimulus matching their expertise” (line 260) as clearly as the authors state.

13. Line 271: Following on from my last point, it would be helpful to report what the different levels of coherence qualitatively stated in this paragraph (i.e., robust, strong, and moderate) mean in quantitative terms by reporting the values in arbitrary units to be able to assess these differences somewhat.

14. Lines 283, 289 & 296: The use of descriptive specifications of levels of significance such as “marginally significantly” and “trended in the predicted direction” have been repeatedly criticized when using the frequentist framework with a dichotomous, albeit arbitrary, cut-off criterion for establishing significance. Although I agree that arbitrary dichotomization of significance of the magnitude of an effect or difference has its downsides, here it would be better to use Bayesian statistics to be able to make statements about the strength of the evidence the data provides for either hypothesis.

15. Pages 13 & 15: It would help the reader in assessing the evidence for the authors’ claims if they were to display data from single participants and not only group averages (Fig. 2) or bar graphs with large error bars (Fig. 4). Illustrating data in simple bar graphs with error bars but without any indication of the spread of the data underlying these figures can hide informative and crucial variability, particularly when comparing groups (Rousselet et al., 2017). This is of major importance in light of the rather small number of participants in each group (12 and 19 participants) and potential individual differences within each group. This could be done by using shaded lines for individual participants in the background of Fig. 2. Using raincloud plots to display the data in Fig. 4 would help to visualize the distributions of the data and the (non-)overlap of these distributions (Allen et al., 2019). Showing the patterns of these data openly, with potentially larger variability for signers at occipital channels, may help to suggest some explanation for the observed results.

16. Line 300: In my eyes the authors are too quick to dismiss the ability to localize different activity patterns. Although EEG does not allow for the localization of activity with the same precision than other techniques (e.g., fMRI), a computational dipole analysis could confirm the claim that activity recorded at frontal electrodes indeed originated from frontal cortex and roughly specify the area of origin. This would be interesting in light of the authors’ claims of higher-level predictions, generated by frontal cortex, playing a crucial role in their study and expertise in general.

Discussion

17. The discussion is in some parts rather speculative and suggests mechanisms that were not explicitly tested in the present data, such as the “frontal cortex is preferentially involved in generating sensory predictions”. Therefore, the presented arguments are not convincingly supporting the interpretation of the findings. Given numerous reports of fontal areas modulating activity in sensory and motor cortices and the expertise at hand being strongly suggestive of top-down mechanisms helping in the interpretation and potentially generation of predictions of the viewed stimuli, I find that the pattern of results at occipital electrodes and the absent top-down modulation of visual cortex raises questions. One possibility that the authors discuss later on is that watching dance actually leads to covertly performing motor imagery of dance movements instead of vocalizing the names of dance routines. If this mechanism were to take place, it should lead to synchronization of frontal with motor or visual cortex or both. Synchrony with visual cortex was not found and synchrony with motor cortex was not tested here. It would have been informative if this synchrony had been formally tested in the present dataset to support this element of the discussion.

18. Particularly, the authors’ claim that domain-general mechanisms would clearly explain the presented findings is not convincing because the only other seriously considered alternative is a language-specific mechanism. Dancing, however, is first and foremost a motor skill for which the names of routines should play a subordinate role during passive watching of routines.

Minor points

1. Line 102: It is honourable to disclose that demographic data was lost during the study, however, to inform the reader as much as possible about sample demographics I suggest to include a rough indication of the participant’s age by specifying that participants were teenagers, college students, adults over the age of 18 or something similar. Otherwise the reader is missing any (even rough) indication about the age of participants that is standard in neuroscience studies.

2. Line 115: Could the white square in the corner of the screen have been a consciously perceived distraction? Please specify the size of the square to indicate the potential for this taking place.

3. Page 15: The presentation of indicators of significance in Fig. 4b is inconsistent with the presentation in panel (a) as this panel is missing indication of the statistical results of the within-subjects results.

References

Allen, M., Poggiali, D., Whitaker, K., Marshall, T. R., & Kievit, R. (2019). Raincloud plots: a multi-platform tool for robust data visualization [version 1; peer review: 2 approved]. Wellcome Open Res, 4(63). https://doi.org/10.12688/wellcomeopenres.15191.1

Bates, D., Mächler, M., Bolker, B. M., & Walker, S. C. (2015). Fitting linear mixed-effects models using lme4. Journal of Statistical Software, 67(1), 1-48. https://doi.org/10.18637/jss.v067.i01

Hentschke, H., & Stüttgen, M. C. (2011). Computation of measures of effect size for neuroscience data sets. European Journal of Neuroscience, 34(12), 1887-1894. https://doi.org/10.1111/j.1460-9568.2011.07902.x

Pernet, C. R., Chauveau, N., Gaspar, C., & Rousselet, G. A. (2011). LIMO EEG: A toolbox for hierarchical linear modeling of electroencephalographic data. Computational Intelligence and Neuroscience, 2011(831409-11). https://doi.org/10.1155/2011/831409

Rousselet, G. A., Pernet, C. R., & Wilcox, R. R. (2017). Beyond differences in means: Robust graphical methods to compare two groups in neuroscience. European Journal of Neuroscience, 46(2), 1738-1748. https://doi.org/10.1111/ejn.13610

Reviewer 2:

general: The topic is interesting and the paper is well written, and the results look convincing. However, I wonder why the dancers show above chance coherence in both dance and sign, whereas signers only show coherence to sign. To speculate, could it be that for the dancers who are (probably) speakers, somehow are able to pickup linguistic cues within the ASL? Furthermore, could the dancers be associating the dance movements with music? Ballet is always accompanied with music, so it could be possible that in addition to motor imagery, musical memory is enhancing the cortical tracking. This may also apply to sign videos. It would be nice to have a section discussing why signers only show coherence to sign, when dancers show coherence to both.

Methods: the dance segments are initially said to be 13 segments (L113) but then in the legend of Fig.1 it is 12.

Why was the video sampling rate increased instead of the EEG being downsampled?

The sign videos are longer than the dance videos. Would this difference affect the coherence?

minor point: In the figure labels, I think it is better to simply put ‘dancers’ and ‘signers’, without non-dancers and non-signers. It is confusing and it is already described in the methods that they are not dancers and signers respectively.
